# Comparison of digital PCR and real time PCR methods for quantitative analysis of African Swine Fever Virus

**DOI:** 10.3389/fvets.2025.1704297

**Published:** 2025-12-10

**Authors:** Silvia Dei Giudici, Piero Bonelli, Maria Giovanna Tilocca, Simona Cau, Pier Paolo Angioi, Anna Maria Sechi, Bruna Vodret, Annalisa Oggiano

**Affiliations:** Istituto Zooprofilattico Sperimentale della Sardegna, Sassari, Italy

**Keywords:** digital PCR, African swine fever virus, quantitative analysis, diagnosis, real time PCR (qPCR)

## Abstract

**Introduction:**

The African swine fever (ASF) is a viral disease of significant socio-economic and animal health impact, commonly diagnosed using molecular techniques. The World Organization of Animal Health (WOAH) has recommended two highly sensitive and specific real time polymerase chain reaction (PCR) procedures (P1 qPCR and P2 qPCR) for the reliable and rapid detection of the ASF virus (ASFV). The present work aimed to standardize two third-generation PCR technologies by using the same WOAH-recommended primer/probe sets.

**Methods:**

We developed two droplet digital PCR protocols ddPCRs and compared their analytical performances to the above-mentioned qPCRs. This involved testing serially diluted plasmid containing the vp72 gene sequence of the ASFV as the template. Clinical positive and negative samples were also analyzed to compare both PCR procedures.

**Results and discussion:**

The ddPCR assays demonstrated excellent linearity (*R*^2^ = 0.999) across a dynamic range from 10^4^ to 1 copies/μl. The limits of detections (LOD) were 3.48 and 2.80 copies/μl and the limit of quantifications (LOQs) were ranging from 25 and 20 copies/μl for the P1 and the P2 procedures, respectively. The LOD values were comparable to those of qPCRs assays. The analysis of the clinical samples evidenced a strong agreement between the qPCR and the ddPCR, with bias values below 0.20, as determined by Bland-Altman analysis. The results of this study indicated that the ddPCR method can be adapted to existing validated qPCR protocols, enabling the quantification of low viral titer samples with similar analytical sensitivity. Moreover, the use of ddPCR allows for the absolute quantification without the need of a calibration curve, providing a reliable tool for ASFV diagnosis and outbreak management. It could also support global effort to control the spread of ASFV. Further validation in diverse matrices (e.g., feed and environmental swabs) could expand its applicability in the One Health frameworks.

## 1 Introduction

The African swine fever (ASF) is a hemorrhagic infectious disease affecting the suid population and transmitted via the African swine fever virus (ASFV), a DNA virus of the Asfarviridae family, genus *Asfavirus* (1–3). The disease is present in four different regions of the world (Africa, Americas, Asia, Europe) in 65 countries, and accounts for the loss of over 2,132,000 animals from January 2022 to 31 June 2025 (4). The polymerase chain reaction (PCR) techniques are widely used to detect the ASFV and are a valuable tool for direct diagnosis. The World Organization of Animal Health (WOAH) has recommended one conventional PCR and two second generation PCR procedures for the reliable detection of the ASFV. The latter two quantitative real time PCR procedures (qPCR) are particularly useful for screening and confirming suspected cases due to their superior sensitivity, specificity, rapidity and high throughput capability. Recently, the advent of third-generation PCR techniques has expanded the possibilities for laboratory diagnosis of infectious diseases. Specifically, the digital PCR (dPCR) is a powerful molecular biological technique used for the precise and absolute quantification of nucleic acids (5, 6). This technique partitions a sample into thousands of individual reactions, allowing theoretically, for the precise target counting at a single-molecule level (7). Unlike qPCR, dPCR relies on endpoint detection and Poisson statistics, thus offering accurate measurement without the need for calibration curves (8–10). In veterinary medicine, dPCR has been successfully applied for the diagnosis of infectious disease in livestock, companion animals, and wildlife (11–14). Its high precision is especially beneficial in animal virology for the detection of persistent or latent infections, such as the viral diarrhea virus (BVDV) (15) and the avian influenza virus (AIV) (16).

The application of dPCR for the detection of the African Swine Fever Virus (ASFV) has shown significant potential in improving the sensitivity and specificity of the viral monitoring, outperforming qPCR in cases of low viral load (17), as often occurs for subclinical infections and environmental samples (18, 19). Furthermore, through the strong multiplexing capability, the dPCR was found to be a valuable tool for the simultaneous distinction of virulent and gene-deleted strains of the ASFV (20, 21).

In this work we standardized two droplet digital PCRs (ddPCR) for the detection and quantification of ASFV using the same primers and probe as described in the WOAH-recommended Taqman qPCR protocols. We detected the optimal primer/probe concentrations and the annealing temperature for both ddPCRs. We then evaluated the linearity, the dynamic range, the limit of detection (LOD) and the quantification (LOQ) of the ddPCRs and compare them to the qPCR ones. Finally, we applied both ddPCRs to the clinical samples of different matrices and compared the performance of the ddPCRs vs. the qPCRs.

## 2 Materials and methods

### 2.1 Experimental design

Two novel droplet digital RT-PCR (ddPCR) protocols were developed for the quantitative detection of the ASFV. They were optimized using the same primers and probe of the TaqMan real time RT-PCR (qPCR) protocols, described by King et al. (22) and Fernández-Pinero et al. (23) and identified respectively as procedure 1 and 2 in the WOAH Terrestrial Manual (24). Both the ddPCRs and the real time PCRs were performed to analyse the DNA standards and the clinical samples. Quantitative detection of the ASFV and the analytical performance of ddPCR and qPCR were compared against each other's.

### 2.2 Preparation of the DNA standards

The DNA standards consisted of serial dilutions of the custom designed plasmid pEX-K4-ASFV-E70p72 (p72 plasmid, Eurofins Genomics, USA), containing the full-length sequence of the p72 gene from the E70 of the ASFV strain (NCBI accession number AY578692.1). A quantity of 85 mg of plasmid was linearized using one unit of EcoRI restriction enzyme (Invitrogen, Thermo Fisher Scientific, Waltham, MA, USA) at 37 °C for 2 h. The digestion was confirmed by 1% agarose gel electrophoresis. The linearized p72 plasmid was denatured at 96 °C for 2 min immediately before the preparation of the two sets of dilutions:

- ° A 10-fold dilution (from 10^6^ to 1 copies/μl) used to prepare the qPCR standard curve and to optimize the ddPCR assay.
- ° A fold-fold dilution from 10^0.7^ copies/μl (5 copies/μl) to 10^0.1^ copies/μl (1.25 copies/μl) and the dilutions 10^1.4^ (25 copies/μl) and 10^1.3^ (20 copies/μl) for both the LOD and the LOQ determination.

### 2.3 Clinical samples

Thirty positive clinical samples made of different matrices (14 spleens, six kidneys, 10 whole blood) were selected from the archive of the Istituto Zooprofilattico Sperimentale (IZS) della Sardegna. The tissue samples were collected from animals naturally infected by the ASFV genotype 1 stored at −80 °C. Thirty negative clinical samples, which were made from the same matrices as the positive samples, were also selected from the archive.

### 2.4 Nucleic acid extraction

The tissue samples (0.5 g) were homogenated at 10% in Phosphate Buffer Saline (PBS) and centrifuged at 1,200 *g* for 10 min. Two hundred μl of supernatant were used for the DNA extraction by MagMax Core Kit (Thermo Fisher, Waltham, MA, USA) in the automated sample preparation workstation MagMax 96 (Thermo Fisher, Waltham, MA, USA), as specified by the manufacturer's instructions.

### 2.5 Primers and probes

The primers and the probes employed for both the ddPCRs and the qPCRs were the same as the ones specified in the WOAH Terrestrial Manual (24) in Procedure 1 (P1) (22), and Procedure 2 (P2) (23). For P2, the alternative probe was used. These assays target different regions of the gene encoding ASFV p72 protein. The primers and probes used are reported in Table 1.

**Table 1:** Primers and probe used for ddPCR and qPCR assays.

**Assay**	**Primers**	**Probe**
Procedure 1 (P1)	CTGCTCATGTATCAATCTTATCGA	6Fam-CCACGGGAGGAATACCAACCCAGTG-TAMRA
	GATACCACAAGATCRGCCGT	
Procedure 2 (P2)	CCCAGGRGATAAAATGACTG	6Fam-TCCTGGCCRACCAAGTGCTT-dark quencher
	CACTRGTTCCCTCCACCGATA	

### 2.6 Taqman real time RT-PCR (qPCR) assays

#### 2.6.1 qPCR protocols

The P1 qPCR assay was performed partly using King et al. procedure with some slight modifications such as the use of the 7500 Fast Real-Time PCR System (Applied Biosystems), the TaqMan Fast Advanced Master Mix (Applied Biosystems) and the addition of 0.8 μM of sense and anti-sense primers and 0.2 μM of TaqMan probe in a total volume of 25 μl containing 5 μl of DNA template. The incubation profile was established as follows: 40 cycles of denaturation at 95 °C for 15 s, annealing at 58 °C for 60 s, after an initial denaturation step at 95 °C for 10 min.

The P2 qPCR assay was performed as per the WOAH Terrestrial Manual, using the TaqMan Master Mix (Roche, Basilea, Switzerland), 0.4 μM of sense and anti-sense primers and 0.1 μM of probe in a total volume of 20 μl containing 2 μl of DNA template. The incubation profile was established as follows: 45 cycles of denaturation at 95 °C for 10 s, annealing at 60 °C for 30 s, after an initial denaturation step at 95 °C for 5 min.

#### 2.6.2 Analytical performances of the qPCRs

The two qPCR protocols, P1 and P2 assays, were performed on DNA standards for analytical performance evaluation. Quantitative linearity of the assays was defined using 10-fold serial dilutions (from 10^6^ to 1 copies/μl) of p72 plasmid and analyzed in duplicate. The average values were used to draw the standard curve and calculate the amplification efficiency and *R*^2^ by 7500 Software SDS v.2.4.1 (Applied Biosystems). The LOD of both assays and the LOQ of P1 qPCR were evaluated using 16 replicates of two-fold serial dilutions of plasmid p72 ranging from 5 to 1.25 copies/μl (from 10^0.7^ to 10^0.1^ copies/μl) and the dilution 1 copy/μl in eight replicates. The LOQ of the P2 qPCR was assessed by analyzing eight replicates of 25 and 20 copies/μl (from 10^1.3^ to 10^1.4^ copies/μl). The LOD at 95% probability was determined via probit regression analysis using SPSS software v. 21 (IBM, USA), whereas the LOQ was set at the lowest dilution showing a coefficient of variation percentage (CV%) below the threshold of 25%, as set by the MIQE Guidelines (6).

#### 2.6.3 Analyses of the clinical samples

The two qPCR protocols, P1 and P2 assays, were used to analyse the clinical samples. The number of the ASFV copies/μl was determined by the interpolation of Cq values from the standard curve. Cq values were calculated using the 7500 Software SDS v.2.4.1 (Applied Biosystems).

### 2.7 Droplet digital RT-PCR (ddPCR) assays

#### 2.7.1 Optimisation of the ddPCR assays

Based on our previous results (data not shown), the dilutions 10^4^ and 10^3^ copies/μl were selected to avoid the saturation of the droplets and optimize the ddPCR assays. Different concentrations of primers and probes in the range between 0.2–0.9 μM (P1 ddPCR) and 0.15–0.25 μM (P2 ddPCR) were tested in eight replicates. Furthermore, to allow an optimal distinction between positive and negative droplets on the same plasmid solutions, the PCR annealing temperature was optimized using a thermal gradient range from 55 °C to 65 °C.

#### 2.7.2 ddPCR protocols

The assays were performed in 20 μl using a 10 μl ddPCRTM Supermix for Probes 2 × (Bio-Rad, Hercules, California, USA) and 2 μl of DNA template for both the P1 and the P2 reactions. As a result of the optimization study carried out on the ddPCR, we used both the primers and the probes at the final concentrations of 0.2/0.8 μM and 0.2/0.9 μM for both the P1 and the P2 ddPCR assays, respectively.

No template controls (NTC) were used for monitoring the primer-dimer formation and contaminations. The ddPCR reaction mix was placed in each well of droplet generator DG8 cartridge (Bio-Rad, Hercules, California, USA) with 70 μl of droplet generator oil (Bio-Rad, Hercules, California, USA). The reaction mix was then emulsified using a QX-200 Droplet Generator (Bio-Rad, Hercules, California, USA). This process allowed for the partitioning of a sample into 20,000 nanoliter sized water-in-oil droplets. Finally, a volume of 40 μl of emulsion was transferred to a 96-well reaction plate (Eppendorf, Hauppauge, NY), heat-sealed with pierceable foil sheets by a PX1TM PCR Plate Sealer (Bio-Rad, Hercules, California, USA) and amplified in a C1000 Touch™ Thermal Cycler (Bio-Rad, Hercules, California, USA).

The cycling conditions for the P1 ddPCR assay were 95 °C for 10 min, followed by 40 cycles at 95 °C for 10 s, then at 58 °C for 60 s and a final cycle at 98 °C for 10 min (to allow for the stabilization of the droplets). The sample can be kept indefinitely at 12 °C. The cycling conditions for the P2 ddPCR assay were 95 °C for 5 min, followed by 45 cycles at 95 °C for 10 s, then at 55 °C for 30 s, 1 cycle at 98 °C for 10 min and infinite 12 °C hold. The annealing temperatures reported in the thermal profile above were chosen based on the optimization study conducted for both ddPCRs assays. A 2.5 °C/s ramp rate was used to ensure each droplet reached the correct temperature for each step of the cycle. At the end of the amplification, the PCR plates were read through the QuantaSoft Droplet Reader (Bio-Rad, Hercules, California, USA) which measures the fluorescence intensity of each droplet and detects their size and shape.

#### 2.7.3 Analytical performances of ddPCR

The two ddPCR protocols, P1 and P2 assays, were used to analyse the standards to evaluate analytical performance. Quantitative linearity of the assays was defined using 10-fold serial dilutions (from 10^4^ to 1 copies/μl) of plasmid p72 analyzed in duplicate. The range of linearity was defined by plotting the log value of plasmid p72 against the log measured value (copies/μl). To evaluate the intra-assay and inter-assay repeatability of both ddPCRs, six different dilutions of plasmid p72 (from 10^4^ to 1 copies/μl) were tested in eight replicates in two different days; the CV% was then calculated and used to assess the repeatability. The LOD was evaluated using 16 replicates of 2-fold serial dilutions of plasmid p72 ranging from 10^0.7^ copies/μl (5 copies/μl) to 10^0.1^ copies/μl (1.25 copies/μl) and the dilution 1 copy/μl in eight replicates. The LOQ of the assays was assessed with the results of the analyses of the eight dilutions from 10^1.3^ (20 copies/μl) to 10^1.4^ copies/μl (25 copies/μl). The LOD and LOQ were determined as previously described for qPCR (see Section 2.6.2).

#### 2.7.4 Analyses of the clinical samples by ddPCR

The two ddPCR protocols, P1 and P2 ddPCR assays, were used to analyse the clinical samples. The clinical samples with a Ct value < 25 in qPCR were diluted before performing ddPCRs to avoid the saturation of the droplets. The absolute concentration of each sample was automatically reported as copy number ASFV/μl with a 95% confidence interval (CI) by the ddPCR QuantaSoft Software V.1.7.4.0917 (Bio-Rad, Hercules, California, USA). Automated threshold settings were applied to analyze the data. The agreement between the qPCR and the ddPCR quantitative results were evaluated via the Bland-Altman plot obtained in Excel.

## 3 Results

### 3.1 Optimization of the ddPCR assays

**Figure 1:**
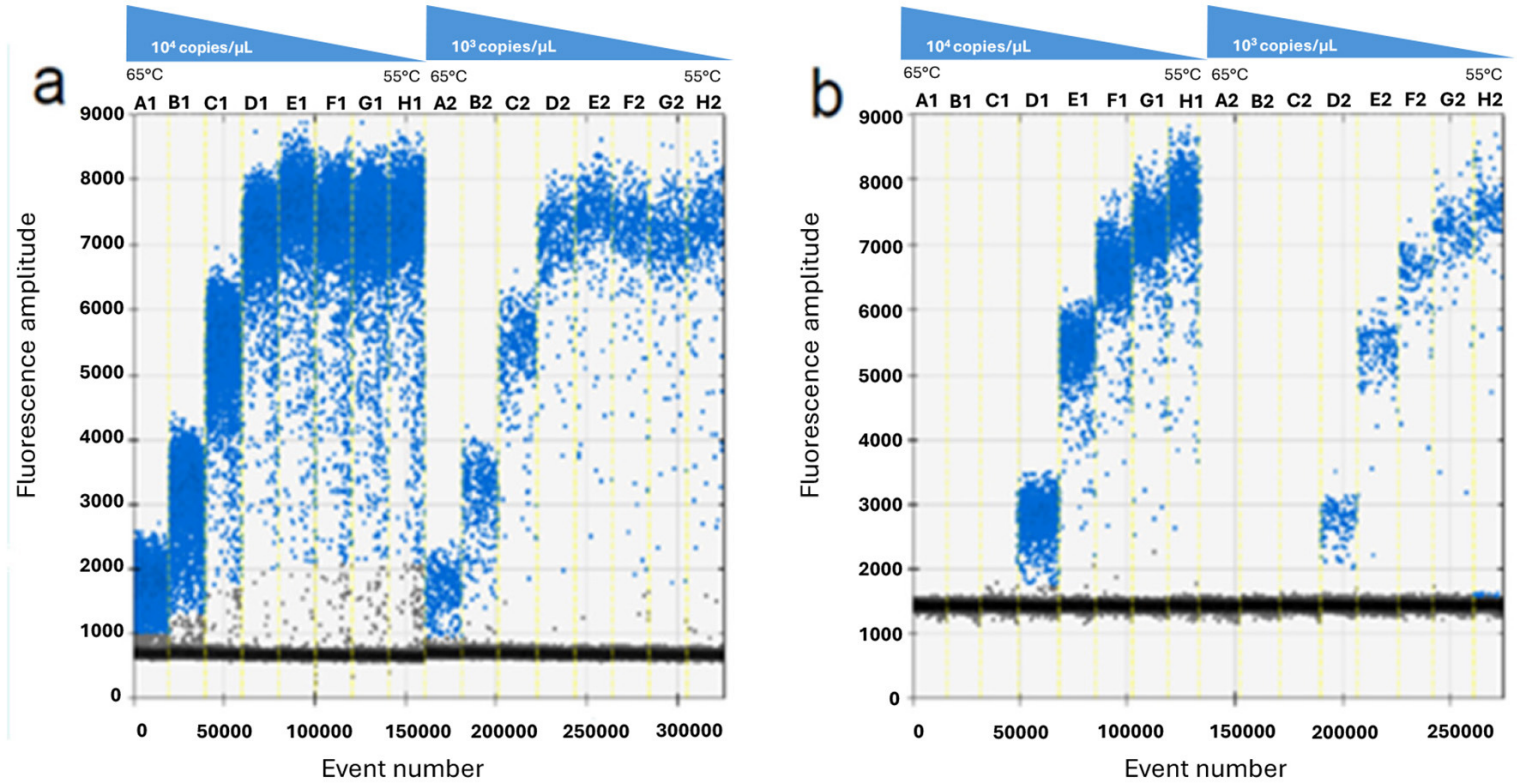
ddPCR optimization of the annealing temperature using a thermal gradient ranging from 65 °C to 55 °C (from A to H) on 10^4^ copies/μl (wells A1–H1) and 10^3^ copies/μl (wells A2–H2) of p72 plasmid. P1 **(a)** and P2 **(b)** ddPCR assays.

**Figure 2:**
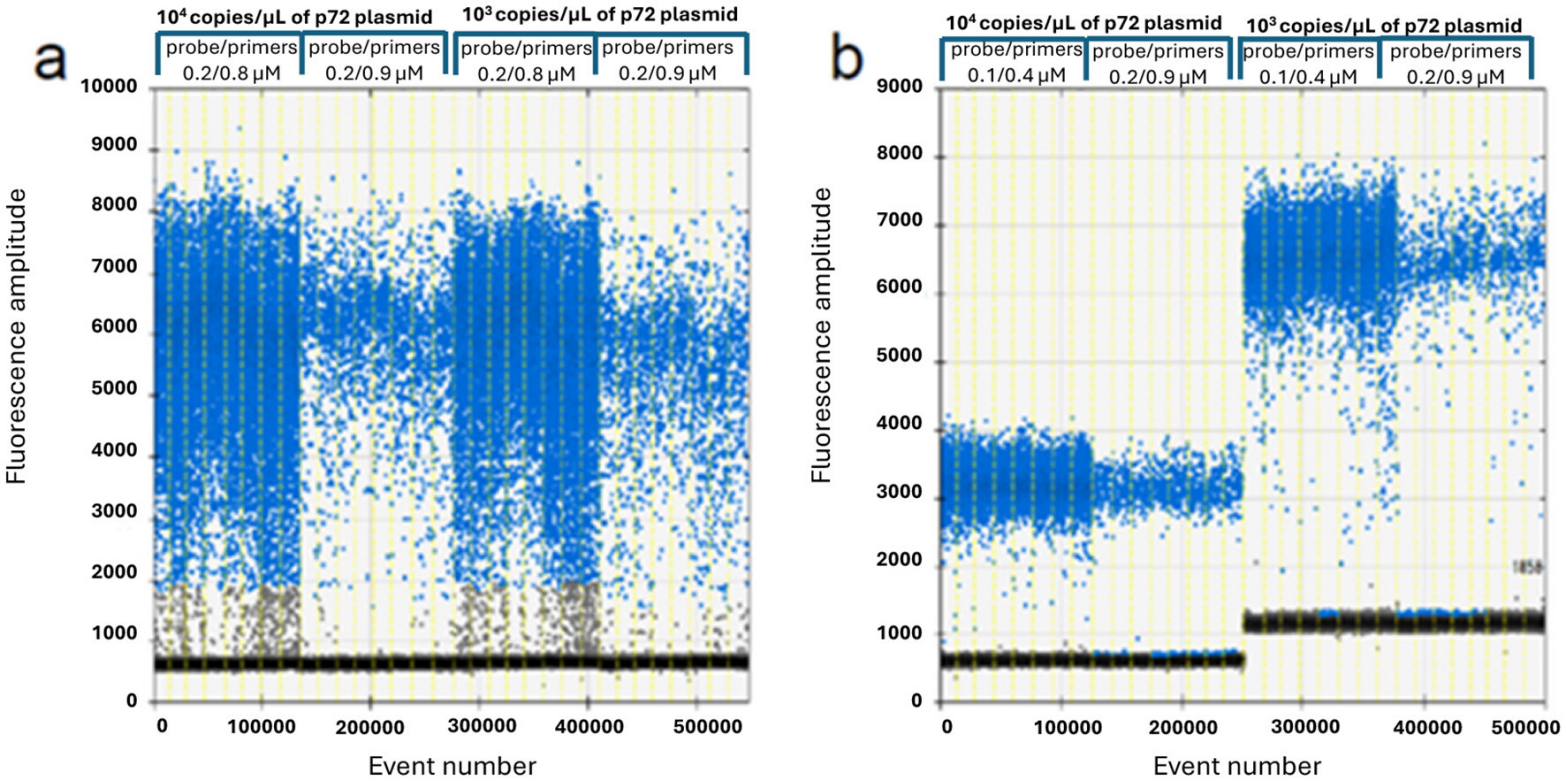
ddPCR primers and probe optimization on 10^4^ and 10^3^ copies/μl of p72 plasmid. P1 assay **(a)**: 0.2/0.8 vs. 0.2/0.9 μM probe/primers concentration; P2 assay **(b)**: 0.1/0.4 vs. 0.2/0.9 μM probe/primers concentration.

The results of the optimization study are illustrated in Figures 1, 2, carried out to determine the appropriate annealing temperatures and the primer/probe concentrations for both the P1 (Figures 1, 2a) and the P2 (Figures 1, 2b) ddPCR assays.

**Figure 3:**
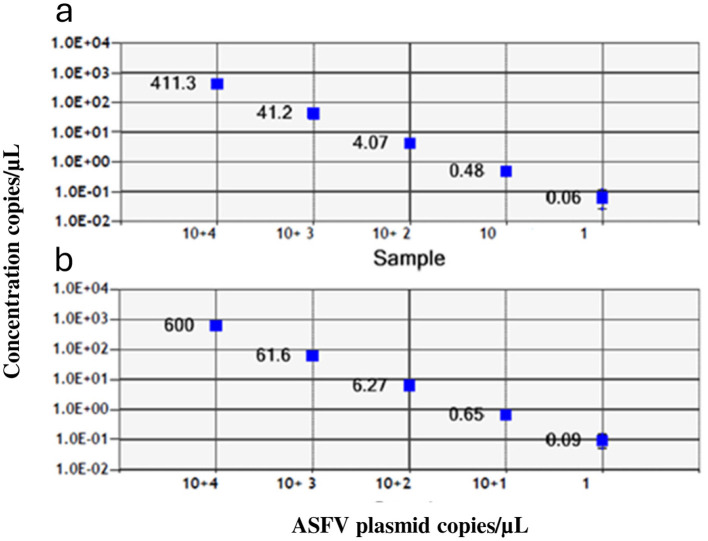
Linearity of the ddPCR P1 **(a)** and P2 **(b)** on serial p72 plasmid dilutions from 10^4^ to 1 copies/μl.

**Figure 4:**
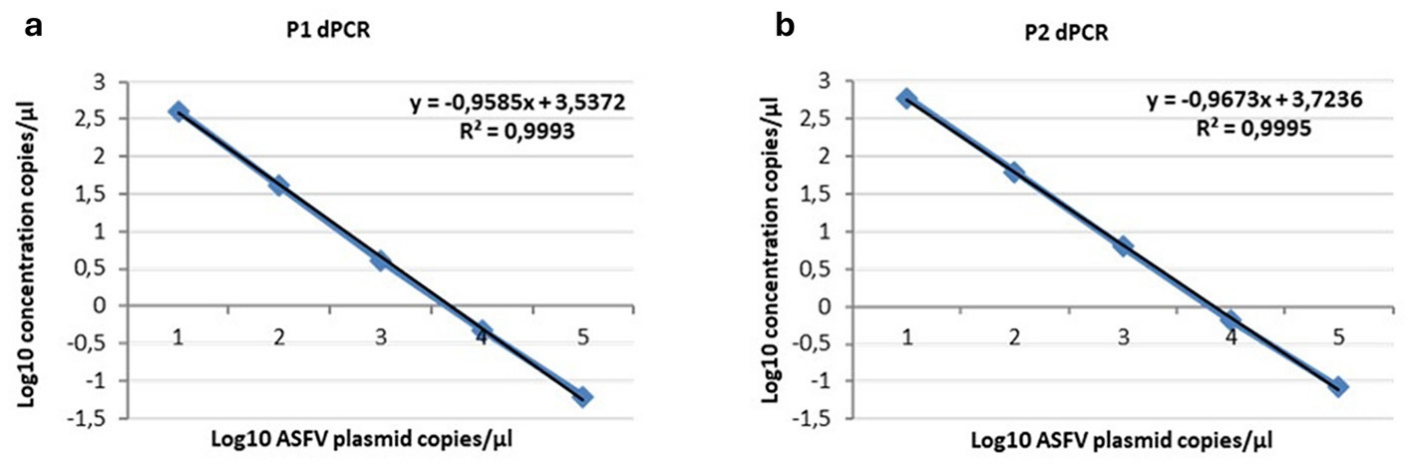
Linearity of the ddPCR P1 **(a)** and P2 **(b)** on serial p72 plasmid dilutions from 10^4^ to 1 copies/μl. Data points for assigned (*x*-axis) and measured values (*y*-axis) were plotted and a trend line was calculated by linear regression.

The optimal annealing temperatures were 58 °C and 55 °C and the primer/probe concentrations were 0.2/0.8 and 0.2/0.9 μM for the P1 and P2 ddPCR assays, respectively. Compared to the qPCR conditions, the P1 ddPCR required no modifications; however, both the annealing temperature and the primer/probe concentration were modified in the P2 ddPCR protocol. In Figures 3, 4 are shown both the linearity and the dynamic range of both ddPCR P1 (Figures 3, 4a) and P2 (Figures 3, 4b).

### 3.2 Analytical performances of ddPCRs and qPCRs

The ddPCR assays showed a good linearity (*R*^2^ = 0.999 for P1 and P2) and a dynamic range between 10^4^ and 1 copies/μl; the qPCRs showed a dynamic range between 10^6^ and 1 copies/μl with both a good linearity (P1: *R*^2^ = 0.998; P2: *R*^2^ = 0.996) and a good efficiency (P1 = 94%; P2 = 92%; Supplementary Figures S1, S2). The results of the copy number detection analysis, obtained by the ddPCR relative to plasmid 10-fold dilutions, gave a good degree of linearity in the range 2–2,000 copies, especially for the P2 ddPCR (Table 2). Furthermore, the P2 ddPCR was able to detect a higher copy number (about 46%) in respect to the P1 assay (Figure 3; Tables 2, 3). To assess the repeatability of the ddPCR assays, the CV% values were considered. The analysis of the eight replicates gave back a CV% lower than the threshold (CV% = 25) for both the ddPCRs in the concentrations 10^2^ and 10 copies/μl for intra-assay and inter-assay, respectively (Table 3).

**Table 2:** p72 plasmid detection of P1 and P2 ddPCRs.

**P72 plasmid copies/2 μl**	**Log_10_ p72 plasmid**	**P1 ddPCR detected copies**	**Log_10_ P1 ddPCR detected copies**	**P2 ddPCR detected copies**	**Log_10_ P2 ddPCR detected copies**
20,000	4.30	8,225.8	3.91	12,006	4.08
2,000	3.30	823.4	2.91	1,232.2	3.09
200	2.30	81.2	1.91	125.2	2.10
20	1.30	9.6	0.98	13	1.11
2	0.30	1.2	0.08	1.7	0.23

**Table 3:** The repeatability of both P1 and P2 ddPCR assays.

**P72 plasmid copies/μl**	**P1 ddPCR intra-assay**		**P1 ddPCR inter-assay**		**P2 ddPCR intra-assay**		**P2 ddPCR inter-assay**	
	**Mean** ±**SD**	**CV%**	**Mean** ±**SD**	**CV%**	**Mean** ±**SD**	**CV%**	**Mean** ±**SD**	**CV%**
10^4^	411.29 ± 31.52	7.66	380.29 ± 43.84	11.53	600.28 ± 25.80	4.30	610.35 ± 14.25	2.33
10^3^	41.17 ± 3.65	8.87	39.32 ± 2.61	6.63	61.61 ± 2.68	4.35	63.97 ± 3.34	5.22
10^2^	4.06 ± 0.44	10.84	4.05 ± 0.02	0.44	6.26 ± 0.84	13.49	6.10 ± 0.23	3.59
10	0.48 ± 0.21	45.86	0.48 ± 0.004	0.92	0.65 ± 0.16	25.10	0.77 ± 0.18	22.81
1	0.06 ± 0.04	73.24	0.08 ± 0.03	33.14	0.08 ± 0.06	68.88	0.07 ± 0.02	31.54

Mean values plus standard deviation of p72 plasmid copies/μl of eight replicates detected by the P1 and P2 ddPCRs.

SD, standard deviation; CV, coefficient of variation.

The LOD values were equal to 3.48 copies/μl (95% CI: 2.57–8.29) of starting plasmid p72 for the P1 ddPCR, 2.80 copies/μl (95% CI: 1.98–13.10) for the P2 ddPCR (Table 4), 1.26 copies/μl (95% CI: 0.97–8.68) for P1 qPCR, and 2.29 copies/μl (95% CI: 1.62–13.87) for P2 pPCR (Table 4, Supplementary Tables S1, S2). The Supplementary Figure S3 depicts the ddPCR and the qPCR LOD values showing the overlap of their 95% confidence intervals. The LOQ was calculated at 20 copies/μl for the P2 ddPCR and 25 copies/μl for the P1 ddPCR (Table 5), 5 copies/μl for the P1 real time PCR (Supplementary Table S1) and 20 copies/μl for the P2 real time PCR (Supplementary Table S2).

**Table 4:** Evaluation of the limit of detection (LOD) of ddPCR and qPCR assays.

**Log_10_ P72 plasmid copies/μl**	**P1 ddPCR+/replicates**	**P1 qPCR+/replicates**	**P2 ddPCR+/replicates**	**P2 qPCR+/replicates**
10^0.7^	16/16	16/16	16/16	16/16
10^0.4^	13/16	16/16	14/16	15/16
10^0.1^	8/16	15/16	14/16	13/16
1	6/8	7/8	5/8	7/8

ddPCR+/replicated, number of ddPCR positive counts/number of replicates analyzed; qPCR+/replicated, number of qPCR positive counts/number of replicates analyzed.

**Table 5:** Evaluation of the limit of quantification (LOQ) of the ddPCR and qPCR assays.

**Log_10_ P72 plasmid copies/μl**	**P1 ddPCR**		**P2 ddPCR**	
	**Mean** ±**SD**	**CV%**	**Mean** ±**SD**	**CV%**
10^1.3^	1.03 ± 0.27	26.33	1.10 ± 0.29	20.78
10^1.4^	0.93 ± 0.17	18.25	1.43 ± 0.22	20.20

Underlined values represent LOQ for the two assays.

CV, coefficient of variation.

### 3.3 Analyses of the clinical samples by ddPCR and qPCR

The quantitative results of the positive clinical samples gathered by both the P1 and the P2 qPCR and ddPCR assays are reported in Supplementary Table S3. The results of each qPCR, expressed in copies of ASFV/μl of DNA sample, were then confronted against their respective ddPCR. The differences between the logarithm (log) of qPCR and ddPCRs quantification values were calculated.

The 30 samples that were negative in qPCRs were also confirmed as negative in both ddPCRs.

In the positive clinical samples, the average log difference was 0.20 (SD = 0.27) for the P1 procedure and −017 (SD = 0.29) for the P2 procedure. For the P1 procedure, the detailed results for tissue and blood EDTA samples were 0.16 (SD = 0.29) and 0.28 (SD = 0.24), respectively. For the P2 procedure, the values were −0.26 (SD = 0.31) for tissue samples and 0.001 (SD = 0.11) for blood EDTA samples.

**Figure 5:**
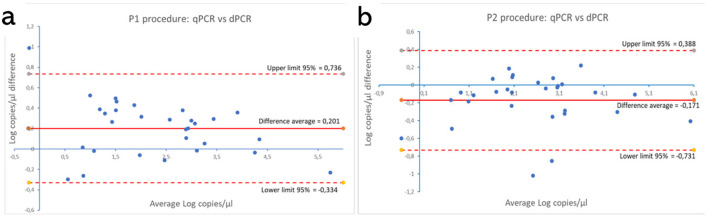
Comparison between both qPCR and ddPCR for the P1 **(a)** and the P2 **(b)** assays using the Bland-Altman plot on 30 clinical samples. The average difference (bias) between the qPCR and the ddPCR methods and the upper and lower 95% CI limits of agreement are indicated in the graph. Numerical values are expressed in log copies/μl.

**Figure 6:**
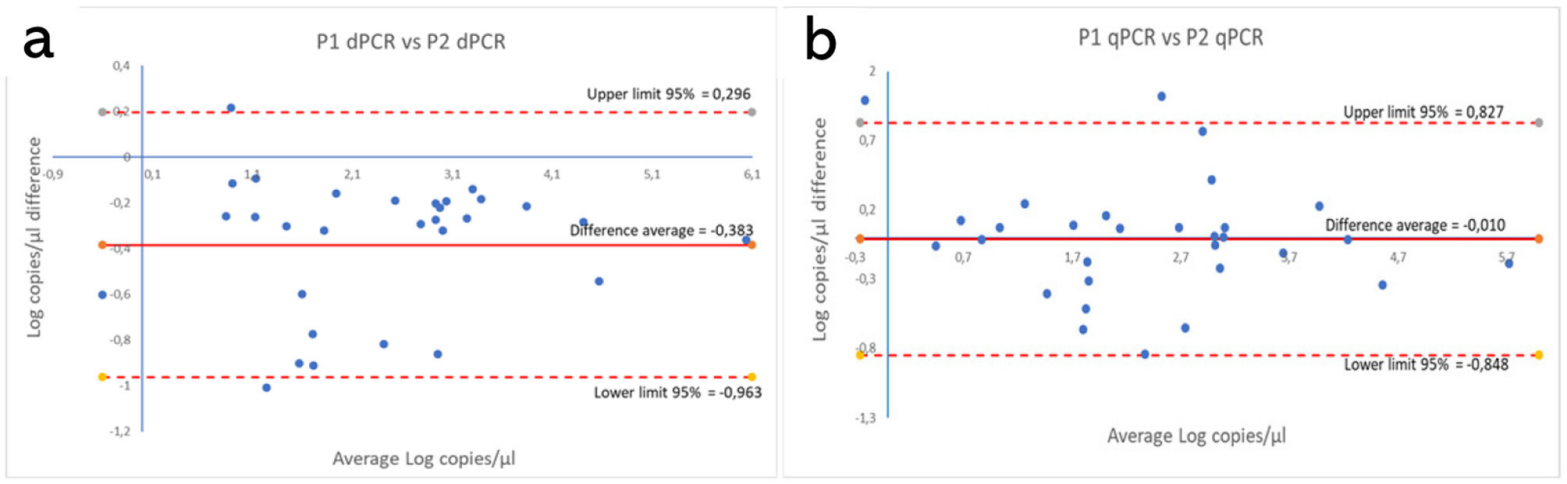
Comparison between both P1 and P2 ddPCRs **(a)** and between the P1 and P2 qPCRs **(b)** using the Bland-Altman plot on 30 clinical samples. The average difference (bias) between the two protocols and the upper and lower 95% limits of agreement are indicated in the graph. Numerical values are expressed in log copies/μl.

These results are shown in Supplementary Tables S3, S4. Figures 5, 6 show the comparison between the qPCRs and the ddPCRs assays using the Bland-Altman plot. The average difference (bias) between the P1 qPCR and the ddPCR was 0.201 log copies/μl (95% = −0.334; 0.736); the difference between the P2 qPCR and the ddPCR was −0.171 log copies/μl (95% = −0.731; 0.388). In contrast, the average difference (bias) between the two ddPCRs was −0.383 log copies/μl (95% = −0.963; 0.296), and the average difference (bias) between the two qPCRs was −0.010 log copies/μl (95% = −0.848; 0.827). By comparing the ddPCRs with the qPCRs, one point (Figure 5a, P1-ddPCR vs. qPCR = 3.33%) or a maximum of two points (Figure 5b, P2-ddPCR vs. qPCR = 6.67%; Figures 6a, b: P1-P2 ddPCR vs. qPCR = 6.66%) fell outside of the upper and lower limits.

## 4 Discussion

This study aimed to standardize two ddPCR assays for the detection and quantification of the ASFV. The standardization process uses the same sets of primers and probe as the P1 qPCR and, for the first time, P2 procedure reported in the WOAH Terrestrial Manual.

The first step in the process was to optimize the primer/probe concentration and the annealing temperature, which are critical factors in determining the efficiency of the dPCR assays (10). The P1 ddPCR did not require any modification compared to the qPCR protocol, whereas the P2 ddPCR required adjustments in both the annealing temperature and the probe concentration. Both assays demonstrated excellent linearity (*R*^2^ = 0.999) across a dynamic range of 10^4^ to 1 copies/μl, though qPCRs exhibited a broader range (10^6^ to 1 copies/μl). The narrower dynamic range of the ddPCR is consistent with prior reports (12, 25), reflecting the technology's reliance on endpoint detection rather than exponential amplification (5, 21). It is noteworthy that the P2 ddPCR detected 46% more copies than the P1, suggesting a higher efficiency, which may be attributable to an enhanced probe design or an improved target accessibility.

The limit of detection for both ddPCR assays (P1: 3.48 copies/μl; P2: 2.80 copies/μl) was found to be marginally higher than that for the qPCRs ones (P1: 1.26; P2: 2.29 copies/μl), despite the 95% confidence intervals overlapping (Supplementary Figure S3). It has been demonstrated by prior research that dPCR exhibits enhanced performance in the detection of rare targets compared to qPCR. This improvement is due to its reliance on Poissonian statistics, its quantitative methodology, and its resilience to biases associated with amplification efficiency (5, 8, 26). In contrast to these findings, our study showed that the sensitivity of digital PCR was comparable to that of quantitative PCR across the methodologies employed. The observation of this increased sensitivity is not due to the lower sensitivity of ddPCR, but rather to the higher sensitivity of qPCR. We could hypothesized that the use of a distinct P1 reaction mixture may have resulted in the observed heightened sensitivity, in line with other researchers (22, 26, 27). The LOD value of our P1 ddPCR is comparable to that of Yang et al. (26), who reported a LOD of 1.97 (1.48–4.12) copies/reaction using the same technology, thereby emphasizing the method's suitability for low-abundance targets, especially in asymptomatic pigs and environmental samples. Conversely, other authors found a higher LOD value (about 30.20 copies/reaction) but employing a nanofluidic chip dPCR platform and with a different primers/probe set (27).

To the best of our knowledge, only a limited number of studies have been published so far, that have evaluated the analytical performances of the P1 (22, 26, 27) and the P2 (23). Whilst comparative studies between dPCR and qPCR are available for the P1 (26), a comparison of the results obtained for the P2 cannot be made. In fact, in the only validation study for the P2 qPCR available in the literature, conducted by Fernández-Pinero et al. (23), the probe used was different to the one used in our tests.

However, the limits of quantification (LOQs) differed: the P2 ddPCR method reliably quantified at 20 copies/μl, whereas the P1 required 25 copies/μl. Intra and inter-assay repeatabilities were robust, with coefficients of variation (CV%) below 25% for concentrations of 10^2^ and 10 copies/μl, respectively. This is in accordance with the MIQE guidelines for dPCR (6), which advocate CV% thresholds of ≤ 25% for accurate quantification. The higher variability observed at very low concentrations (e.g. 1 copy/μl) was expected due to the well-documented limitation of dPCR stochastic partitioning effects (28).

The analyses of the clinical samples (spleen, kidney, whole blood) performed using both methods evidenced a very good agreement between both the qPCR and the ddPCR, with bias values below 0.20. Similar observations can be made when comparing the P1 and P2 protocols, employing either ddPCR, which yielded a bias value of 0.38 or qPCR which produced almost coincident results (bias = 0.01).

This study demonstrated that ddPCR is a robust alternative to qPCR for the ASFV detection, particularly in low-concentration samples. The optimized P1 and P2 ddPCRs provide reliable tools for diagnostics and outbreak management, supporting global efforts to control the ASFV spread. Quantitative assays for ASFV detection may also find use for vaccine research, to determine immune response following experimental infection, being a more effective tool to detect low viral loads. While ddPCR offers absolute quantification without standards, its higher cost and throughput limitations may restrict widespread adoption in resource-limited settings. However, we should point out that not only would the costs of qPCR be similar or even higher when applied to a small number of samples, but also that technological innovation will inevitably lead to a reduction in the costs of ddPCR. Moreover, its high sensitivity and further validation in diverse matrices (e.g., feed and environmental swabs) could expand its applicability in the One Health frameworks.

## Data Availability

The original contributions presented in the study are included in the article/Supplementary material, further inquiries can be directed to the corresponding author.
