# Supplementary material for: Comparison of digital PCR and real time PCR methods for quantitative analysis of African Swine Fever Virus
Source: Front Vet Sci. 2025 Dec 10;12:1704297. doi: 10.3389/fvets.2025.1704297 (PMC12730159; doi:10.3389/fvets.2025.1704297)
Supplement: Supplementary file 1 [file Supplementary_file_1.zip › Supplementary Material/Supplementary Data Sheet 1.DOCX]

SUPPLEMENTARY FIGURES


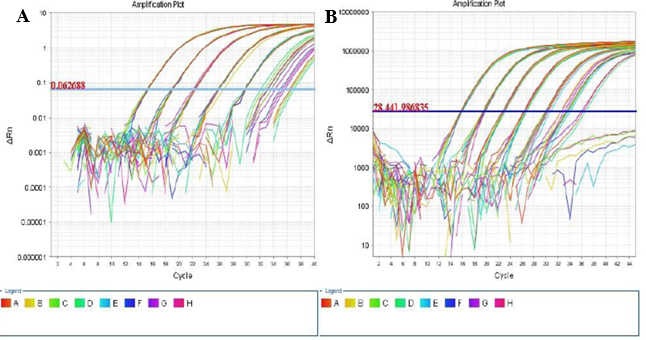


Figure S1. Amplification plot of qPCR P1 (a) and P2 (b) on serial p72 plasmid dilutions from 10^+6^ to 1 copies/μl. ALT text: qPCR amplification plots demonstrating that both assays P1 (a) and P2 (b) successfully detect the p72 gene across a wide range of concentrations, from 10^+6^ to 1 copies/μl.


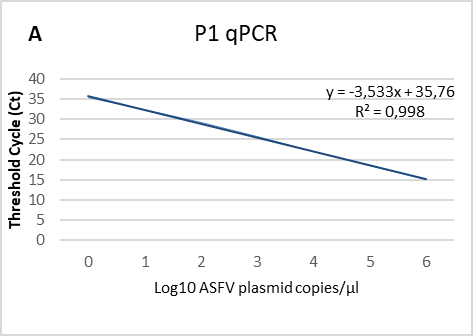

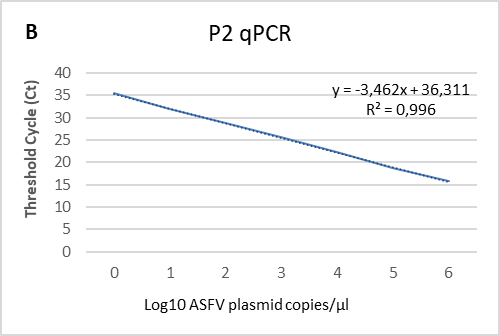


Figure S2. Linearity of qPCR P1 (a) and P2 (b) on serial p72 plasmid dilutions from 10^+6^ to 1 copies/μl. ALT text: linear standard curves confirm that the qPCR assays P1 (a) and P2 (b) provide reliable and quantitative measurements of the p72 plasmid over the entire 6-log dilution series.

Figure S3: Limit of detection at 95% confidence interval (LOD, 95% CI) of P1 and P2 ddPCR and qPCR assays. ALT text: comparison of the assays' sensitivity, showing the lowest concentration each method (ddPCR vs. qPCR) can reliably detect. ddPCR demonstrates a lower Limit of Detection (LOD) for both assays P1 and P2.

SUPPLEMENTARY TABLES

Table S1. Limit of quantification (LoQ) of P1 qPCR.

^a^Mean values plus standard deviation of p72 plasmid copies /μl of 16 replicates detected by P1 and P2 Real qPCRs. ^b^Coefficient of variation. Underlined value represents the CV% corresponding to LOQ.

| **Log10 P72 plasmid Copies/µl** | **P1 qPCR**  **Mean values**  **Copies/µl ±SD^a^** | **CV%^b^** | **P2 qPCR**  **Mean values Copies/µl ±SD^a^** | **CV%^b^** |
| --- | --- | --- | --- | --- |
| 10 ^0.7^ | 4,55 ± 0,91 | 20,00 | 6,05 ± 2,92 | 48,26 |
| 10 ^0.4^ | 2,82 ± 1,06 | 37,58 | 2,12 ± 0,90 | 42,45 |
| 10 ^0.1^ | 1,66 ± 0,51 | 30,72 | 1,65 ± 1,12 | 67,88 |

Table S2. Limit of quantification (LoQ) of P2 qPCR.

^a^Mean values plus standard deviation of p72 plasmid copies /μl of 8 replicates detected by P1 and P2 Real qPCRs. ^b^Coefficient of variation. Underlined value represents the CV% corresponding to LOQ.

| **Log10 P72 plasmid Copies/µl** | **P2 qPCR Mean values Copies/µl ±SD^a^** | **CV%^b^** |
| --- | --- | --- |
| 10 ^1.3^ | 18,23±3,15 | 17,16 |
| 10 ^1.4^ | 18,65±4,49 | 24,07 |

Table S3. Comparison of qPCR and ddPCR assays for quantitative detection of ASFV in positive clinical samples.

| **Sample ID** | **P1 qPCR copies/µl** | **P1 ddPCR copies/µl** | **Log differences** | **P2 qPCR copies/µl** | **P2 ddPCR copies/µl** | **Log differences** |
| --- | --- | --- | --- | --- | --- | --- |
| 40853 S6 | 1561.73 | 880.00 | 0.25 | 604.86 | 1374.7 | -0.36 |
| 40853 S9 | 1929.04 | 1713.30 | 0.05 | 330.96 | 2376.7 | -0.86 |
| 51534 S1 | 88.75 | 102.00 | -0.06 | 616.87 | 673 | -0.04 |
| 51534 S2 | 259.94 | 337.00 | -0.11 | 1169.44 | 2456.7 | -0.32 |
| 51534 S5 | 427528.06 | 730000.00 | -0.23 | 658902.62 | 1680000 | -0.41 |
| 61072 S | 24622.38 | 19800.00 | 0.09 | 54016 | 69270 | -0.11 |
| 53410 S | 49.99 | 21.00 | 0.38 | 102.68 | 125.3 | -0.09 |
| 81588 S | 1088.13 | 684.00 | 0.20 | 104.38 | 1092 | -1.02 |
| 83673 S | 24.32 | 10.00 | 0.39 | 14.02 | 18.3 | -0.12 |
| 87326 S | 1.94 | 0.20 | 0.99 | 0.2 | 0.8 | -0.60 |
| 152 S | 11.69 | 12.23 | -0.02 | 9.95 | 15.2 | -0.18 |
| 81505 S | 2.59 | 5.13 | -0.30 | 2.98 | 9.3 | -0.49 |
| 22653 S1 | 36.8 | 20.00 | 0.26 | 121.39 | 79.33 | 0.18 |
| 22653 S2 | 29.24 | 13.20 | 0.35 | 136 | 105.67 | 0.11 |
| 51534 K1 | 1385.92 | 732.00 | 0.28 | 1187.71 | 1220 | -0.01 |
| 51534 K2 | 894.75 | 699.00 | 0.11 | 1493.71 | 1471 | 0.01 |
| 51534 K3 | 978.26 | 630.70 | 0.19 | 1116.67 | 1190.7 | -0.03 |
| 51534 K4 | 1253.73 | 1304.00 | -0.02 | 1244.42 | 2413.3 | -0.29 |
| 53410 K | 56.27 | 18.00 | 0.50 | 85.15 | 147 | -0.24 |
| 61072 K | 17150.21 | 18670.00 | -0.04 | 17745.68 | 35870 | -0.31 |
| WB1 | 12366.13 | 5453.33 | 0.36 | 7351.01 | 8946.67 | -0.09 |
| WB2 | 1036.23 | 433.33 | 0.38 | 1024.02 | 855.33 | 0.08 |
| WB3 | 122.9 | 46.00 | 0.43 | 85.89 | 96.33 | -0.05 |
| WB4 | 7.29 | 7.07 | 0.01 | 7.59 | 9.2 | -0.08 |
| WB5 | 18.37 | 5.50 | 0.52 | 46.76 | 56 | -0.08 |
| WB6 | 3877.71 | 1976.67 | 0.29 | 5035.15 | 3030 | 0.22 |
| WB7 | 523.19 | 271.00 | 0.29 | 446.71 | 420.33 | 0.03 |
| WB8 | 56.97 | 19.67 | 0.46 | 46.48 | 39.67 | 0.07 |
| WB9 | 5.43 | 10.00 | -0.27 | 4.11 | 6.1 | -0.17 |
| WB10 | 148.39 | 72.00 | 0.31 | 127.78 | 104.3 | 0.09 |
| Average (SD) |  |  | 0.20 (0.27) |  |  | -0.17 (0.29) |
